# Imaging of Bladder Cancer: Standard Applications and Future Trends

**DOI:** 10.3390/medicina57030220

**Published:** 2021-03-01

**Authors:** Rasha Taha Abouelkheir, Abdalla Abdelhamid, Mohamed Abou El-Ghar, Tarek El-Diasty

**Affiliations:** Radiology Department, Urology and Nephrology Center, Mansoura University, Mansoura 35516, Egypt; Abdallahshady333@yahoo.com (A.A.); maboelghar@yahoo.com (M.A.E.-G.); teldiasty@hotmail.com (T.E.-D.)

**Keywords:** bladder, cancer, imaging, CT, MRI, diagnosis

## Abstract

The evolution in imaging has had an increasing role in the diagnosis, staging and follow up of bladder cancer. Conventional cystoscopy is crucial in the diagnosis of bladder cancer. However, a cystoscopic procedure cannot always depict carcinoma in situ (CIS) or differentiate benign from malignant tumors prior to biopsy. This review will discuss the standard application, novel imaging modalities and their additive role in patients with bladder cancer. Staging can be performed with CT, but distinguishing between T1 and T2 BCa (bladder cancer) cannot be assessed. MRI can distinguish muscle-invasive from non-muscle-invasive tumors with accurate local staging. Vesical Imaging-Reporting and Data System (VI-RADS) score is a new diagnostic modality used for the prediction of tumor aggressiveness and therapeutic response. Bone scintigraphy is recommended in patients with muscle-invasive BCa with suspected bony metastases. CT shows low sensitivity for nodal staging; however, PET (Positron Emission Tomography)/CT is superior and highly recommended for restaging and determining therapeutic effect. PET/MRI is a new imaging technique in bladder cancer imaging and its role is promising. Texture analysis has shown significant steps in discriminating low-grade from high-grade bladder cancer. Radiomics could be a reliable method for quantitative assessment of the muscle invasion of bladder cancer.

## 1. Introduction

Bladder cancer (BCa) is the second most common urogenital malignancy, preceded by prostate cancer. It is considered the 10th most widespread cancer in the world [1,2]. The incidence of bladder cancer rises with age and is ~3–4 times higher in men. Cigarette smoking is the most important risk factor [3]. Urothelial carcinoma represents about 90% of bladder cancer cases and typically presents with multifocality and recurrence; other subtypes are squamous cell carcinoma (6–8%) and adenocarcinoma [4].

Bladder cancer clinical management strategies and prognosis depend on the extent of loco-regional disease and on the discrimination between superficial (≤T1 stage) and muscle-invasive disease (≥T2 stage). Non-muscle-invasive (superficial) bladder cancer is managed with transurethral resection of the bladder tumor with or without intravesical or photodynamic chemotherapeutic adjuvants [5], while muscle-invasive bladder cancer is managed with radical cystectomy, irradiation, chemotherapeutic adjuvants, or a combination [6].

The idea of this review is to discuss and differentiate the standard imaging modalities, and the novel techniques in bladder cancer imaging. We will focus on the advantages and limitations of each imaging method and their role in discriminating muscle-invasive from non-muscle-invasive disease as well as lymph node involvement prior to operative management.

Painless hematuria is the typical symptom of BCa [7]. The first diagnostic workup of bladder cancer includes clinical examination, urine analysis, ultrasound (US) or cystoscopy, followed by diagnostic transurethral resection of the bladder tumor (TURBT) and histopathology [1,8].

Although bladder cancer diagnosis is histopathologically based via cystoscopy and biopsy, cystoscopy cannot reliably determine the depth of bladder muscle infiltration and development of metastasis [9], so precise staging is pivotal. Finding the best imaging modality to use for tumor staging prior to operative intervention remains controversial. To optimize the diagnosis of bladder cancer, novel diagnostic imaging procedures are being discussed for the accurate use of each modality in its place.

## 2. Imaging Considerations of Urinary Bladder Cancer

### 2.1. Plain X-ray

**Figure 1:**
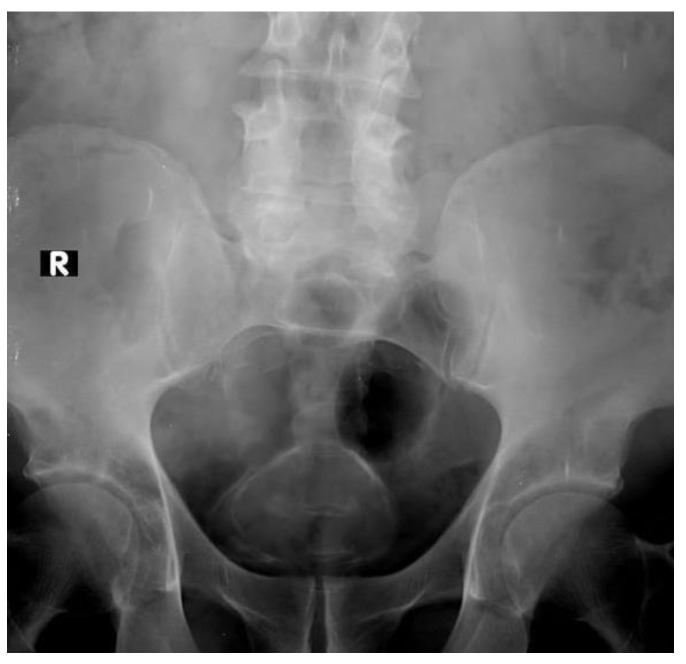
Continuous curved line of calcification of bilharzial bladder wall (R mark: right side).

In approximately 1% of bladder cancers, urothelial or squamous cell carcinoma, calcification is visible on plain film. It can be focal, linear, punctuating or coarse. A plain X-ray may suggest the diagnosis for bilharzial bladder, which is complicated by carcinoma. The calcification occurs as a continuous curved line of calcification of the bilharzial bladder wall (Figure 1). The continuation of the linear calcification is disrupted if bladder cancer develops [10]. Tuberculous calcification of the bladder wall is rare and seen only after healing [11], but when present, a faint irregular rim of calcium may outline the bladder wall. By the time it become more extensive, there may be associated calcification in the seminal vesicles [12].

The dystrophic calcification infrequently occurs with the necrotic nature of the bladder tumor. It can be seen as fine speckles of calcification or as a dense calcified nodule within the bladder [10].

### 2.2. Intravenous Urography “IVU”

**Figure 2:**
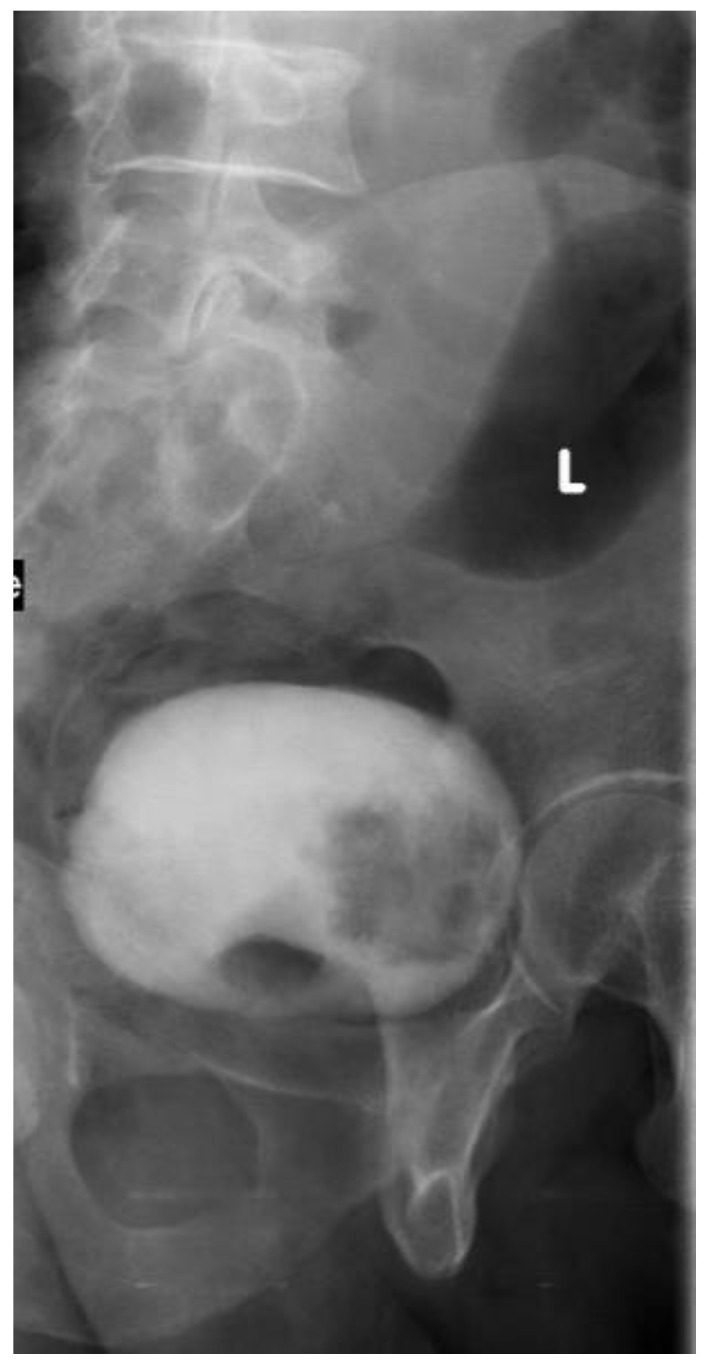
IV urography: full bladder film, left oblique view: a large filling defect at the bladder base and left lateral wall (L mark: left oblique). (Pathologically proven to be urothelial carcinoma of the bladder).

In the delayed phase of IVU (cystogram), fungating tumors within the bladder appear as filling defects. However papillary tumors may be missed on IVU due to the masking effect of contrast material in a full bladder and in the post void phase (Figure 2). Diverticular tumors may not be visualized on IVU. Exposure to ionizing radiation is a considerable risk of IVU, equal to a 0.1% incidence of radiation-induced carcinoma [13]. Renal failure due to contrast nephropathy has been documented in 0.8% of cases with no prior complaint of renal disease [13]. Furthermore, severe contrast-induced reactions occur in 0.22% of the ionic and 0.04% of the nonionic contrast material studies [14].

The recommendations of the First International Consultation of Bladder Tumors (FICBT) state that IVU is not of significant value in the detection of bladder cancer [15]. Palou and colleagues reported that the incidence of synchronous tumor in the upper tract was less than 2.0%, but rose to 7.5% in tumors arising at the trigone [16]. So, the International Bladder Cancer Group (IBCG) only advise imaging of the upper tract in selected cases with multicentric tumors or tumors arising at the trigone [17].

Only 26% to 86% of bladder cancer could be detected with IVU. Furthermore, its reliability in detecting small cancer is still controversial, ranging from 0.5–1 cm as a lower size limit of sensitivity. Conventional urography cannot differentiate the masses of vesical origin from those of extravesical origin infiltrating the bladder as well as lymph node evaluation, therefore CT urography has been proved superior due to soft tissue and nodal discrimination in the CT examination [18].

### 2.3. Ultrasonography

Bladder tumor detection by ultrasound relies upon the size and site of the tumor. Tumors less than 0.5 cm in size and tumors at the bladder neck are hard to delineate. However, a higher detection rate with an accuracy of 95% can be obtained for tumors >0.5 cm located on the lateral or posterior bladder walls [19].

#### 2.3.1. Conventional Ultrasonography

The ultrasound appearance of a bladder tumor may be an intraluminal nonmobile mass or localized thickened bladder wall [20].

#### 2.3.2. Color Flow Doppler Ultrasonography

**Figure 3:**
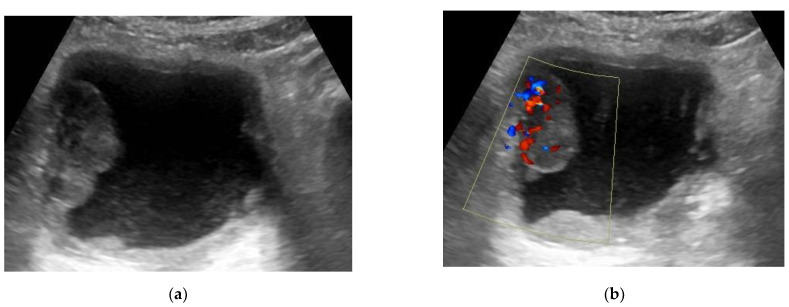
(**a**) Transverse US (ultrasound) image of the bladder showing a large well-defined echogenic mass (4 × 2 × 3.7 cm) at the right lateral bladder wall. (**b**) Color Doppler ultrasound detected the presence of vascularity within this mass. (This mass was pathologically proven to be urothelial carcinoma, stage T2 N0).

The flow within the mass can be assessed with Doppler ultrasound and thus can distinguish the tumor from a blood clot. A bladder tumor appears as an echogenic soft-tissue lesion projecting inside the lumen and adherent to the bladder wall. The existence of tumoral blood flow does not assess the stage or aggressiveness of tumor. Ultrasound has shown little value in the staging of bladder cancer (Figure 3) [21].

#### 2.3.3. Contrast-Enhanced Sonography

Many authors studied the usefulness of contrast-enhanced ultrasonography on different organs in the assessment of the vascular nature of tissue and for overcoming some of the limitations in tumor characterization with gray-scale and Doppler ultrasound examination [22].

At gray-scale US, the bladder wall appears homogeneously hyperechogenic. However, the bladder wall layers can be differentiated with the use of contrast-enhanced sonography [22]. It also has an added value in differentiating high from low grade cancer using contrast-sonographic perfusion curves [23].

#### 2.3.4. Three-Dimensional US

Developments in image reconstruction have made three-dimensional (3D) US possible. This provides additional information including a 3D display of different pathologies. It allows BCa scanning in different planes and increasing the sensitivity of diagnosis. It also allows the distinction of the superficial disease from the infiltrative disease [24].

### 2.4. CT Urography

**Figure 4:**
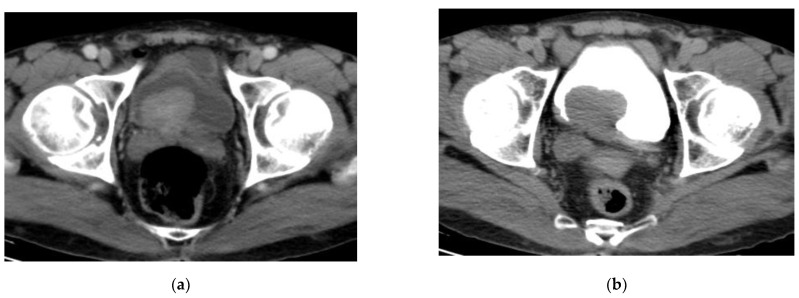
CT urography: (**a**) nephrographic phase: an enhancing intravesical soft tissue mass at the posterior bladder wall; (**b**) excretory phase: washout of contrast medium. The bladder mass is recognizable as a filling defect inside the opacified urine. (Urothelial carcinoma, stage T2 N0).

CT urography is the most frequently used imaging technique for detection and staging of bladder cancer with a diagnostic accuracy estimated of 91% in detection and 35–55% in staging [25,26]. The examination is often done with a three-phase protocol: noncontrast, nephrographic and delayed phases [27]. Bladder cancer appears as an intraluminal polypoidal or nodular mass or localized wall thickening. The bladder mass exhibits variable degrees of postcontrast enhancement at the nephrographic phase, performed 100–120 s. after contrast medium injection. The tumoral mass shows washout and can be detected as a soft tissue filling defect in the contrast material-filled hyperattenuating lumen on delayed phase of CT urography [28,29] (Figure 4).

Perivesical fat infiltration by the tumor can be detected on CT cystography [30]. However, CT cannot accurately assess the degree of invasiveness of the muscle wall, i.e., the discrimination of stage T1 from stage T2 or stage T2a from stage T2b, but it can differentiate stage T3b or higher stage tumors with an accuracy of about 83–93% [31].

***T staging classification of bladder tumor:*** the American Joint Committee on Cancer (AJCC) TNM system is the most widely used staging system [32].

T0: no evidence of tumor.
Ta: noninvasive papillary carcinoma.
Tis: Carcinoma in situ.
T1: Tumor invades the subepithelial connective tissue (lamina propria).
T2: Tumor invades the musclularis propria bladder wall
T2a: Tumor invades the superficial muscle (inner half)
T2b: Tumor invades the deep muscle (outer half)
T3: Tumor invades the perivesical tissue
T3a: Microscopic.
T3b: Macroscopic. (extravesical tumor)
T4: Tumor invades any of the following: prostate, uterus, vagina, pelvic wall, and abdominal wall
T4a: Tumor invades the prostate, uterus, or vagina
T4b: Tumor invades the pelvic or abdominal wall.

Bladder cancer spreads initially to pelvic lymph nodes, then common iliac and retroperitoneal lymph nodes [20]. Lymph node (LN) staging accuracy by CT depends on the size and shape of the affected LN and is considered significant on CT if the short axis is more than 8 and 10 mm in pelvis and abdomen, respectively [33]. Overstaging in about 30% of patients can occur with reactive lymph nodes more than 10 mm in short axis. On the other hand, metastasis in lymph nodes with the short axis less than 8 mm can be missed [34].

Overall, performing a CT study is crucial as the initial investigation of hematuria. Imaging of the upper tract is essential in cases with bladder cancer due to increased incidence of multicentric tumors (2% of cases diagnosed as bladder cancer have an upper tract tumor) [35]. In addition, in the follow up protocol of patients, assessment of the upper tract is crucial to assess metachronous tumor in the upper tract [27].

### 2.5. Virtual Cystoscopy (VC)

Conventional cystoscopy (CC) remains the ideal technique used for detection, accurate assessment and follow up of bladder cancer [36]. The creation of virtual reality imaging can be obtained via 3D volume rendering methods with rapid acquisition with further interactive intraluminal navigation through organs with lumens, like urinary bladder [37,38]. Vining and his coauthors were the first to apply this modality in bladder cancer detection [39]. With the development of MDCT and many studies later, virtual cystoscopy has been established [40,41].

Two different techniques of virtual cystoscopy: [42]

1. Noncontrast CT of the pelvis subsequent to air/CO_2_ filling via a urethral catheter, then virtual reconstruction of images.
2. Delayed phase of MDCT urography, then CT virtual cystoscopy reconstruction.

Virtual cystoscopy is processed with the volume rendering technique. Navigation through the bladder and processing of 3D virtual reality images can be performed with the camera of V.C. The viewing scope of the radiologist can be performed in 360 degrees in different directions inside the bladder (CT cystoscopy) to evaluate the urinary bladder wall and lumen. When any abnormal lesion is detected, it must be fully assessed in all projections.

Bladder distension is necessary for performing CT virtual cystoscopy. The air insufflation technique is the best for adequate bladder distension, due to the maximum different attenuation gradients being obtainable between the bladder wall and the lumen distended with air, about 1000 H.U., that reduces the image artifacts [43]. In a study done by Teama A.H. et al., CT virtual cystoscopy detected 91/93 tumors that were detected by conventional cystoscopy with sensitivity (97.8%): 38 tumors were polypoidal (38/39) and 53 were sessile (53/54) [42].

**Figure 5:**
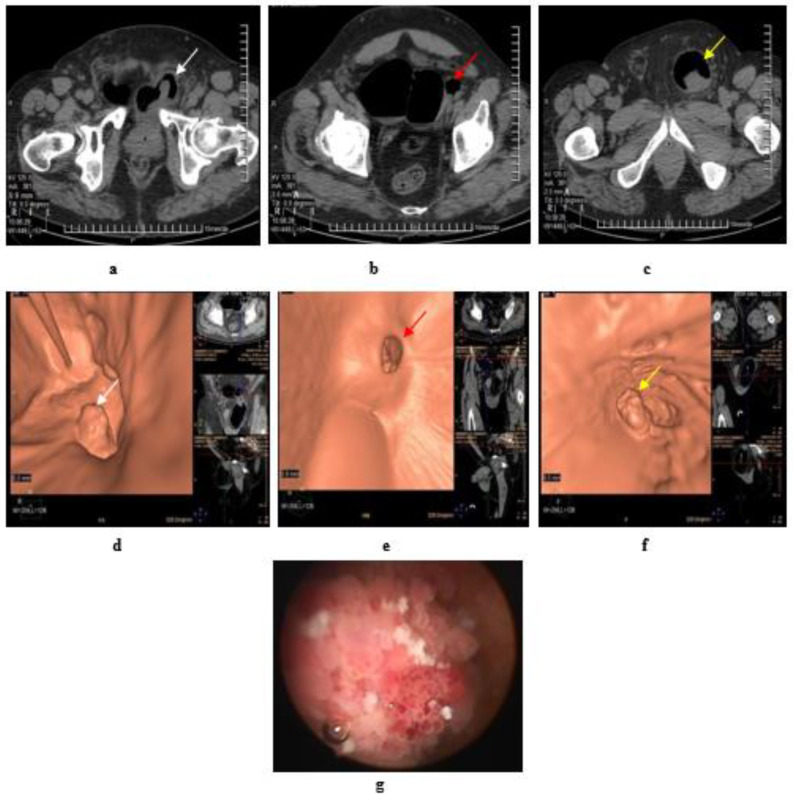
Papillary urothelial carcinoma of the bladder. (**a**) Axial MDCT of the pelvis after air insufflation: intravesical soft tissue mass (white arrow) at left posterolateral wall. (**b**) A large right bladder diverticulum with a wide neck and a small left diverticulum showed two small intradiverticular soft tissue masses (red arrow). (**c**) A large left inguino-scrotal hernia with a large cystocele inside and a soft tissue mass within the cystocele (yellow arrow). (**d**) Virtual CT cystoscopy of the intravesical mass noted at left posterolateral bladder wall, that was referred to at (a) (white arrow). (**e**) Virtual CT cystoscopy of two small soft tissue masses inside a small left side diverticulum, that was referred to at (b) (red arrow) (**f**) Virtual CT cystoscopy of a large soft tissue mass at the cystocele arising from its posterior wall, that was referred to at (c) (yellow arrow). (**g**) Conventional cystoscopy confirms the presence of intravesical masses.

**Figure 6:**
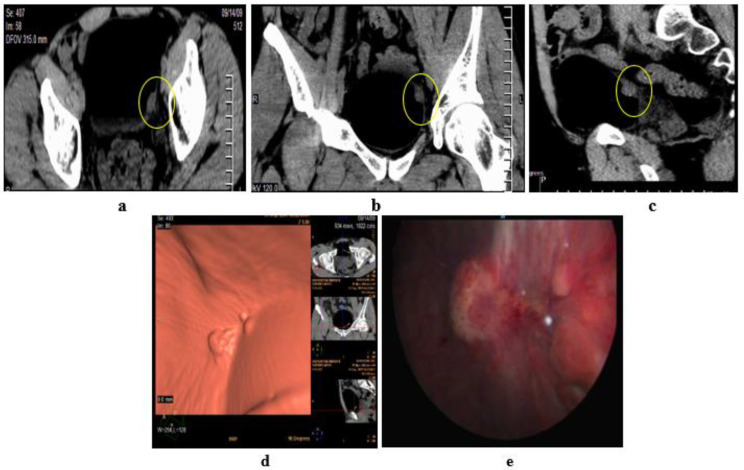
Papillary urothelial cell carcinoma of the bladder. (**a**–**c**) Axial, coronal, sagittal images: only one lesion at the left lateral bladder wall. (**d**) CT virtual cystoscopic volume rendering image: 3 small lesions at the left lateral bladder wall. (**e**) Conventional cystoscopic image confirms the presence of the three lesions at the left lateral bladder wall [42].

Virtual cystoscopy has many merits over conventional cystoscopy. It is minimally invasive, enabling the bladder to be totally visualized intraluminally. It also permits navigation to sites that cannot be accessed by conventional cystoscopy and precisely measures the accurate tumor dimensions (Figure 5 and Figure 6). CT virtual cystoscopy has also limits: it cannot detect color changes of the mucosa. Tissue biopsy also cannot be obtained. Virtual cystoscopy can be of great value as a complementary tool when conventional cystoscopy is not feasible or if there is a contraindication to its use [42].

### 2.6. Magnetic Resonance Imaging

The superiority of MRI in the staging of bladder cancer is due to its increased soft tissue contrast resolution [4]. Functional assessment using diffusion-weighted imaging (DWI) and dynamic contrast enhanced (DCE), can add further data about tumor invasiveness and infiltration to the surrounding structures, especially in distinguishing muscle-invasive bladder cancer (MIBC) from non-muscle-invasive bladder cancer (non-MIBC) [44,45].

The multiplanar capability of MRI leads to minimizing the partial volume and optimizing imaging when evaluating the degree of muscle layer invasiveness. Coronal images can help with better assessment of tumors at the lateral bladder wall and dome, however sagittal images can delineate tumors at the anterior and posterior wall and dome [4].

Three Tesla (3 T) MRI show a more highly advanced performance than 1.5 Tesla (1.5 T) MRI due to the high-resolution difference between the tumor and normal tissues, and the assessment of the degree of invasion [46]. On the other hand, MRI can overestimate the degree of depth of bladder wall invasion post-transurethral tumor resection or chemo-radiotherapy [47].

#### 2.6.1. High-Resolution T2-Weighted Imaging (T2WI)

T2WI is the imaging modality of choice for bladder cancer staging, due to its ability to detect the depth of the tumor infiltration. Superficial tumors display intermediate to high SI on T2WI with an intact adherent hypointense muscle wall. On the other hand, muscle-invasive tumors interrupt the hypointense line of the detrusor muscle [48].

#### 2.6.2. Diffusion-Weighted Imaging (DWI)

DWI depends on the Brownian movement of free-water molecules. Tumors with high cellular density lead to the diffusion restriction of water molecules and show a high SI on the DWI. Quantitative assessment with the apparent diffusion coefficient (ADC) map measures the degree of restricted water diffusion with the measurement of the ADC value that is reduced with increasing tumor aggressiveness [49,50]. Advances in DWI are promising for improving the detection and staging of bladder cancer with nodal infiltration. Combined use of T2WI with DWI have shown higher sensitivity, specificity and accuracy compared to T2WI alone, thus can be effectively used in differentiating muscle-invasive from non-muscle-invasive tumors [50,51].

Normalized ADC (nADC) of the bladder tumor, calculated by ADC(tumor)/ADC (reference tissue) using the urine and surrounding muscle tissue as a reference, has shown superior sensitivity compared to ADC alone in assessment of the aggressiveness of bladder cancer, according to a study done by Wang HJ and his colleagues on thirty patients with histolopathologically proven bladder cancer [52].

#### 2.6.3. Dynamic Contrast-Enhanced (DCE) Imaging

DCE-MRI significance assessing tumor aggressiveness depends upon the tumor neo-angiogenesis. Malignant tumors with hypervascularity are enhanced earlier on DCE-MRI [53,54]. Many studies have reported variable accuracy of DCE-MRI in differentiating muscle-invasive and non-muscle-invasive bladder cancer as well as between organ-confined and non-organ-confined cancers, ranging from 85–97% to 82–84%, respectively [53,55].

With dynamic contrast studies, the tumor, bladder mucosa, and submucosa exhibit early enhancement, however the bladder wall muscle keeps its hypointense SI with delayed enhancement. The most widely used staging system according to Takeuchi et al. [51,56]:

Stage T1: Intact muscle layer adjacent to the tumor and displays hypointense SI at high resolution T2W sequence without early enhancement on dynamic study.
Stage T2: Interrupted hypointense line of the muscle layer with early enhancement of the tumor with no extravesical fat invasion.
Stage T3: irregular outer boundary of the tumor with distortion of the extravesical fat with the same SI of the tumor.
Stage T4: Tumor infiltrates the surrounding organs or pelvic walls.

#### 2.6.4. Time-SI Curves

The time-SI curve is generated by drawing a region of interest and measuring the SI value at the area of maximum enhancement within the tumor [57].

Type I (ascending) curve: early tumoral enhancement, with sequential slow ascent.
Type II (plateau) curve: early tumoral enhancement, then a plateau.
Type III (descending) curve: early tumoral enhancement, then washout.

The formation of new vascularity (neo-angiogenesis) is an essential factor in tumor growth Thus, inhibition of angiogenesis can cause suppression of tumor growth [58]. Several studies have reported a positive correlation between intratumoral microvessel density (MVD), “a measure of tumour angiogenesis” and the bladder cancer progression [59]. Imaging is an essential step of diagnosis, staging and follow up of bladder cancer. DCE-MRI can evaluate the neo-angiogenic stage of bladder cancer through analysis of the pattern of enhancement [60].

**Figure 7:**
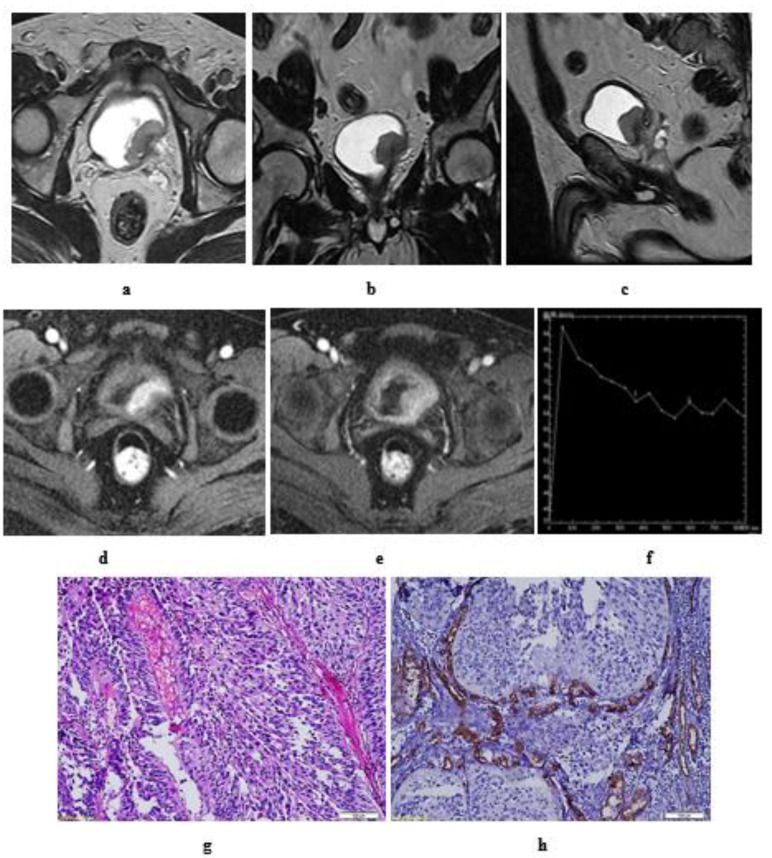
Grade III papillary urothelial carcinoma of the bladder. Stage T2: in a male patient, aged 52 years. Hematuria was his main complaint without prior TURBT (transurethral resection of bladder tumor). MRI T2 high resolution: axial (**a**), coronal (**b**), and sagittal (**c**) image: an intravesical soft tissue mass, with intermediate SI, at left posterolateral bladder wall, near the bladder neck and left ureteric opening. Disrupted low SI line of the muscle layer of the bladder wall. DCE- MRI: axial (**d**) early postcontrast T1 wi: The tumor is seen enhancing earlier than the muscle layer of the bladder wall. (**e**) Late postcontrast T1 wi: delayed enhancement of the detrusor muscle which is seen interrupted by the tumor. No perivesical fat infiltration. (**f**) Time-SI curve: early enhancement of the mass followed by washout slope. Stage T2. (**g**) hematoxylin-eosin stained slide: grade III papillary urothelial carcinoma. Stage T2. (**h**) CD34 expression (×250): mean of MVD = 68.

Hassanien O.A. et al. studied the correlation between dynamic MRI and bladder tumor angiogenesis using CD34 (endothelial cell marker) to detect the tumor microvessel density (Figure 7) [56]. He found that there was a reasonable positive correlation between the washout slope of the dynamic CE-MRI curve and mean MVD (r = 0.471, *p* = 0.001) that coincided with the results reported by Tuncbilek et al. [61]. He found also angiogenesis increased in parallel with increasing tumor stage and grade, which was completely consonant with the results of Gehani et al. [59] and that also confirmed that MVD in bladder carcinoma correlated with the tumor grade, stage, and its malignant potential.

#### 2.6.5. Inch Worm Sign

In 2009, Takeuchi et al. [51] described the inchworm sign that was discovered in fungating tumors with a connective tissue stalk, the stalk appears as arch-like, the tumor displays hyperintense SI overlying the connective tissue stalk that displays hypointense SI. This configuration represents the stage T1 or less. Tumor aggressiveness can be predicted with this appearance as an imaging biomarker.

**Figure 8:**
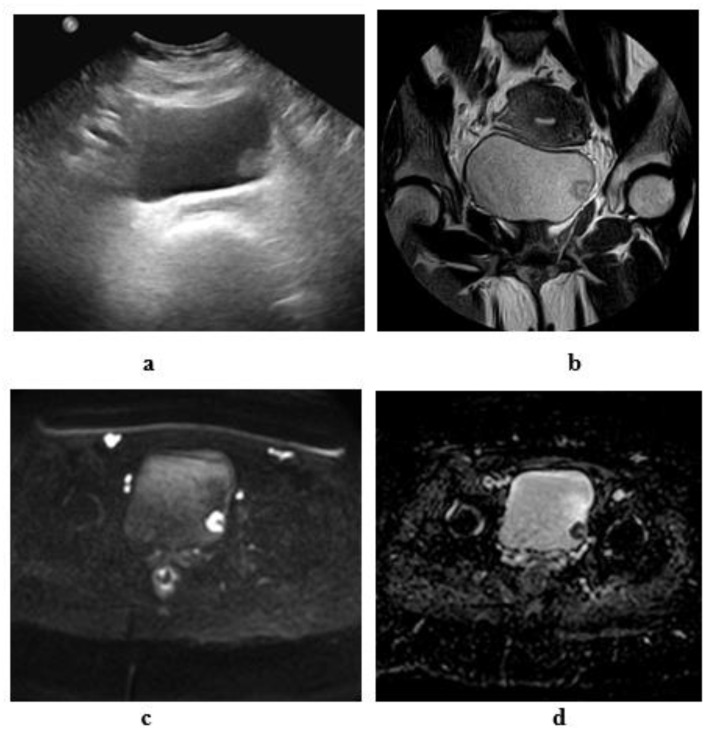
Stage T1 urothelial carcinoma: (**a**) transverse ultrasound image: an echogenic soft tissue mass at the left lateral wall. (**b**) A high resolution coronal T2 MRI: superficial soft tissue mass at the left lateral wall with arch- shaped high SI tumor covering the low SI submucosal stalk (inch worm sign). (**c**): DW-MRI (diffusion-weighted magnetic resonance imaging) (b value = 1400): showing restricted signals of the tumor and not the submucosal stalk. (**d**): ADC (apparent diffusion coefficient) map: the tumor displays hypointense SI and hyperintense SI of the submucosal stalk. Note the intact muscle wall of the urinary bladder. (ADC value = 1 × 10^−3^ mm^2^/s).

However, the connective tissue stalk is characteristic for T1 or less bladder cancer. It was reported that some cases of stage T2 or more represented with “inchworm sign” on diffusion-weighted magnetic resonance imaging (DW-MRI) [62,63] (Figure 8). So, DWI findings and high-resolution T2 w sequences must be correlated.

## 3. Staging of Urinary Bladder Cancer: TNM Guidelines

A multiparametric (mp) MRI, including morphological T2-weighted imaging (T2WI) together with the added value of functional sequences of diffusion-weighted and dynamic contrast-enhanced imaging are of great importance in terms of the local staging of primary tumors, nodal affection, distant metastasis, detection of recurrence and assessing the therapeutic response [64,65].

The increased capability of MRI in the field of local staging using morphologic sequences as T2-weighted images (T2WI) together with functional diffusion-weighted images (DWI) / apparent diffusion coefficient (ADC map), and dynamic contrast-enhanced MRI (DCE), allow for the identification of tumor site, size, and morphology, thus decreasing the false staging assessment using T2 high resolution sequence alone [2].

On T2WI, BCa usually has a signal intensity (SI) intermediate to the muscle. The detrusor muscle displays low SI on T2WI, and when stage T2 tumor develops, it appears interrupted [66,67]. On DWI, BCa appears low on the ADC map, and restricted diffusion [27].

### 3.1. T-Staging

**Figure 9:**
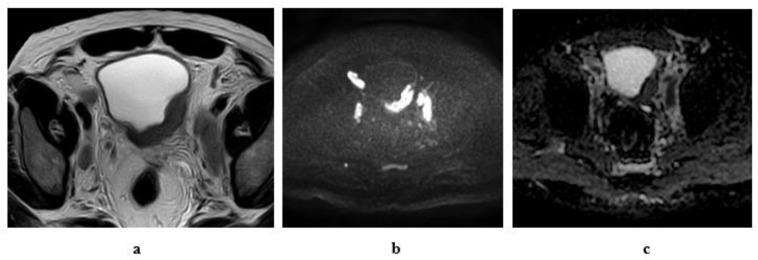
A 55-year-old male patient with an intravesical mass at the left posterolateral bladder wall (Micropapillary urothelial carcinoma, stage T3b N2): (**a**) high resolution T2 MRI: the soft tissue mass has an intermediate SI with infiltration of the perivesical fat, bilateral external and internal iliac lymphadenopathy. (**b**) Diffusion w MRI: b value 1400: restricted signals of the mass and bilateral LNs. (**c**) ADC of the mass = 0.8 × 10^−3^ mm^2^/s, of the lymph nodes = 0.9 × 10^−3^ mm^2^/s.

**Figure 10:**
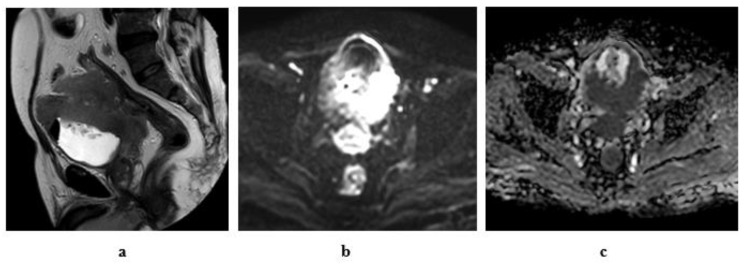
A 61-year-old male patient with stage T4a N2 urothelial carcinoma: (**a**) high resolution T2 MRI: an intravesical mass at the posterior bladder wall infiltrating the seminal vesicles as well as the anterior wall of the rectum. (**b**) Diffusion w MRI (**b** value 1400): restricted signals of the mass at the posterior bladder wall as well as the infiltrated portion of the anterior wall of the rectum and bilateral external iliac LNs. (**c**) ADC of the mass = 0.7 × 10^−3^ mm^2^/s.

The European Association of Urology (EAU) guidelines state that the gold standard diagnostic tool of BCa staging is TURBT. However, an unprofessional resection results in the absence of the detrusor layer in the biopsy and may cause tumor understaging [68]. MRI can overcome these limitations with high staging accuracy. mpMRI is a significant imaging tool for distinguishing non-muscle-invasive from MIBC, as well as the diagnosing of T3 and T4 disease (Figure 9 and Figure 10). Furthermore, it can specify the muscle-invasive tumor suitable for bladder-sparing therapy and chemo or radiotherapy, from others for the surgical planning [4].

### 3.2. N-Staging

A lymph node more than 8 mm in the short axis is considered significant, together with changes in its morphology. The morphologic criteria of LN involvement include a rounded configuration (reviewed on ≥2 perpendicular planes to avoid false positive results), the loss of hilar fat, in addition to contour irregularities [69].

Lymph node assessment with MRI is of limited accuracy. Size is not specific in detection of nodal micrometastases, and more than 90% of normal-sized metastatic LNs in bladder cancer have s short axis diameter ≤5 mm, Nodal reactive hyperplasia will also increase the false-positive results [70].

Several studies reported that diffusion-weighted imaging has shown promising results in differentiating benign from malignant nodal affection [4]. A wide range of ADC values exists in patients with metastatic abdominal lymph nodes, with a tendency of higher ADC values in benign lymph nodes [71].

The quantification of the apparent diffusion coefficient (ADC) of nodes by DW-MRI is showing substantial promise for nodal characterization, although the relatively poor re-producibility of ADC measurements of smaller lymph nodes (<5 mm) remains a limitation [71].

### 3.3. M-Staging

The commonest metastatic organs are liver, bone and lungs in descending order [72]. In cases with muscle-invasive disease, CECT of the chest, abdomen, and pelvis is highly advised for the picking up of lymph node and distant metastasis.

Isotope bone scanning is still considered the most sensitive imaging modality in bone metastatic detection [73]. Although MRI has a superior sensitivity and specificity for bony metastasis detection [74], it has not been integrated into the recent guidelines because of limited reading experience, and lower cost effectiveness [75].

18F-fluorodeoxyglucose (FDG)-PET is of limited significance in the local staging of bladder cancer. Although, several studies recommend its use in the detection of the distant metastasis of primary tumors as well as recurrent disease, recent guidelines do not support its routine use in BCa [76].

## 4. Intradiverticular Carcinoma

**Figure 11:**
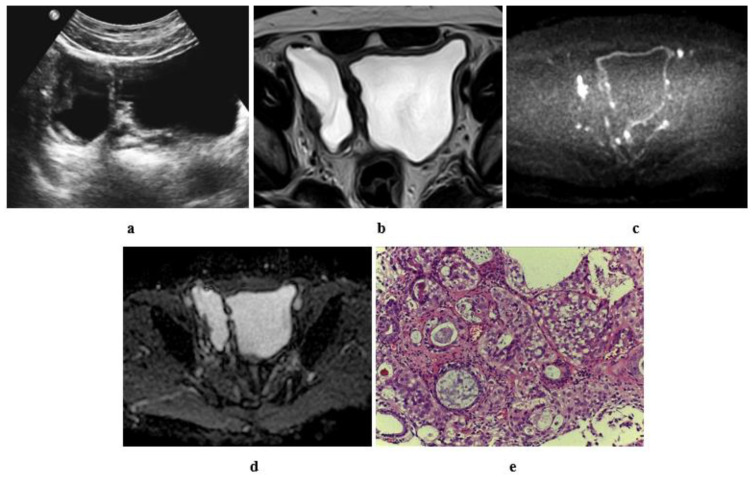
A 60-year-old male patient with stage T2 urothelial carcinoma (**a**) ultrasonographic image of right side bladder diverticulum with intradiverticular soft tissue lesion. (**b**) High resolution T2 w MRI, axial image: intradiverticular mass with intermediate SI. (**c**) Axial diffusion MRI ((**b**) value = 1400): restricted diffusion of the intradiverticular mass. (**d**) ADC of the mass with hypointense SI: 1 × 10^−3^ mm^2^/s. (**e**) Histopathologic image: GIII urothelial carcinoma.

The relationship between urinary bladder diverticula and bladder cancer is established. The incidence of primary intradiverticular tumors ranges from 1–10% [77]. The commonest malignant tumor type arising within vesical diverticula is urothelial carcinoma (78%) (Figure 11), followed by squamous cell carcinoma (17%) [78].

Urinary stasis causes irritation of the bladder mucosa and prolonged bladder affection with carcinogens inside urine. Consequently, it predisposes to malignant transformation of the urothelial lining of the diverticula [77]. Most of publications state that complete tumor resection and strict follow up may provide success in the management of intradiverticular cancers [79].

## 5. Ureterocele Urothelial Carcinoma

Ureterocele is a pseudocystic dilation of the distal portion of the pelvic ureter. It protrudes into the bladder lumen. It is very rare to develop urothelial cancer within an ureterocele [80]. Ultrasound has a significant role in the detection and initial diagnosis of intraureterocele tumors. Irregular echogenicity of the intravesical cystic structure and the absence of acoustic shadowing suggests the presence of an intraureterocele tumor [81].

## 6. Mimics of Primary Urothelial Carcinoma

### 6.1. Neoplasms Mimicking Urothelial Carcinoma

#### 6.1.1. Urachal Carcinoma

Primary urachal adenocarcinomas are very uncommon, representing only 0.5–2% of vesical malignant tumors, however about 34% of adenocarcinomas of the bladder are urachal in origin [82].

**Figure 12:**
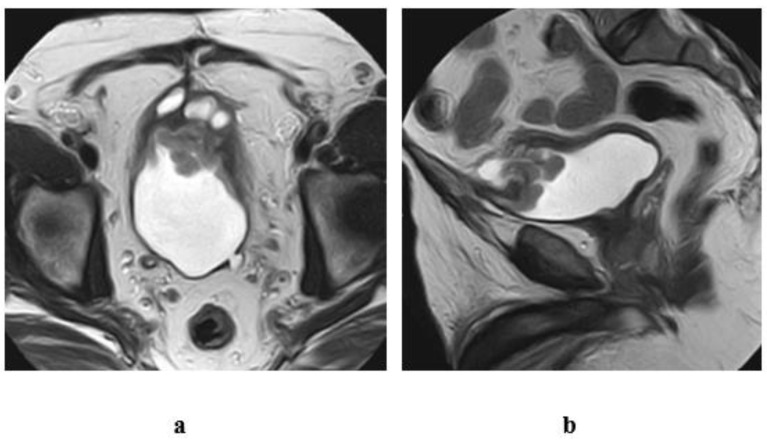
A case of urachal adenocarcinoma (enteric type) in a 60-year-old male patient, complaining of hematuria. T2 w MRI of the pelvis (**a**) axial, (**b**) sagittal views: inhomogenous midline mass of mixed cystic and soft tissue components, infiltrating the dome of the bladder and connected superiorly to the umbilicus with a track displaying hypointense SI, representing the fibrous remnants of urachus.

The urachal carcinoma appears on US as a hypo- or anechoic complex cystic mass superior to the bladder. On CT, it is usually seen as a midline mass, near water attenuation, anterior and superior to the bladder dome. It may be solid, cystic, or mixed. Low-attenuation components represent mucin contents [83]. On MR imaging, it appears as a supravesical mass with increased signal intensity on T2-weighted images due to the presence of the mucin component (Figure 12) [84]. There is calcification in 50–70% of cases and it is considered nearly pathognomonic for urachal adenocarcinoma [83].

#### 6.1.2. Lymphoma

Primary lymphoma of the bladder is rare [85]. The secondary involvement of the bladder by systemic lymphoma is more common. There are no distinct imaging features to discriminate bladder lymphoma from urothelial cancer [86]. However, bladder lymphoma appears as a lobulated mass with vascularity on color Doppler US and enhancement on CT or MRI [87].

#### 6.1.3. Paraganglioma

Bladder paraganglioma is often indistinguishable from urothelial carcinoma. However it is characterized by intense enhancement on postcontrast CT or MRI, or the presence of necrosis or hemorrhage within the mass. Clinical presentation is important [88].

#### 6.1.4. Metastasis

Bladder metastasis appears as solitary or multiple vascular nodular masses. However, a multifocal appearance and the patient history are significant [87].

### 6.2. Benign Lesions That Can Mimic Urothelial Carcinoma

#### 6.2.1. Cystitis

Acute cystitis appears as bladder wall thickening, oedema and perivesical fat distortion. Infrequently, bladder cancer can cause diffuse bladder wall thickening as well as hyperemia, resembling chronic cystitis. Th correlation of symptoms with patient history is helpful in distinguishing cystitis from bladder cancer.

**Figure 13:**
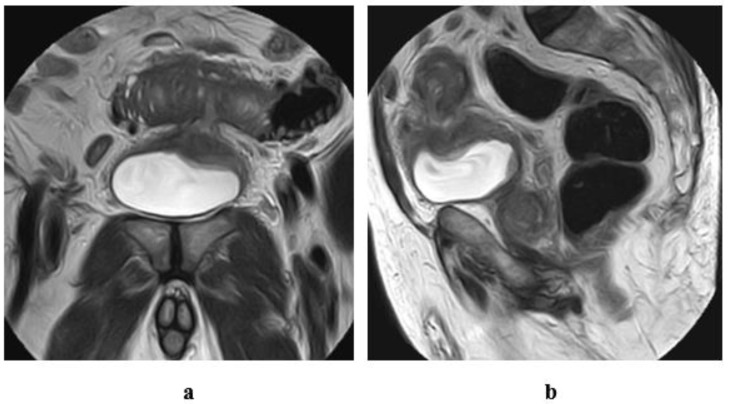
A case of eosinophilic cystitis secondary to intestinal diverticulitis and subsequent vesicointestinal fistulous communication, in a 77-year-old male patient. T2 w MRI of the pelvis (**a**) coronal, (**b**) sagittal views: localized thickening of and a defect at the dome of the bladder, associated with track formation extending from the dome to the bowel loop superior to the bladder. Inflammatory reaction and diverticulitis of the related portion of the intestinal loop.

Eosinophilic cystitis is a rare disease simulating bladder cancer. The cause remains unclear. It is characterized by inflammation, mainly by eosinophils, throughout all the layers of the bladder wall. Typically, most of the patients present with irritative bladder symptoms and a bladder wall thickening and/or a large bladder mass simulating bladder carcinoma (Figure 13) [89].

#### 6.2.2. Cystitis Glandularis

Cystitis glandularis is a common chronic inflammatory disorder in which transitional cells have undergone glandular metaplasia [90]. Masses from cystitis glandularis appear as filling defects at IVU. On CT or MRI, it appears as an enhancing polypoid soft tissue mass. It displays low SI on T1-WI and low signal intensity with a central branching high signal intensity on T2-WI. The hyperintense central area represents the vascular connective tissue stalk [91]. The muscle layer should be intact, a feature that discriminates cystitis glandularis from malignant carcinoma [85].

#### 6.2.3. Endometriosis

On ultrasound, a bladder endometrioma more commonly appears as a hypoechogenic mass that mimics bladder cancer. The echogenic bladder wall mostly surrounds it, while bladder cancer often does not have this boundary [92]. On T1-weighted MRI imaging, the hyperintense foci, due to the presence of chronic blood products, help to differentiate an endometrioma from bladder cancer [93].

#### 6.2.4. Tuberculosis

Bladder tuberculosis is most often represented by an irregular asymmetric bladder wall. Later, marked fibrosis results in a marked contraction and small capacity of the bladder, “thimble bladder” [94].

## 7. Recurrence

Transurethral resection of the tumor remains the surgical mainstay for the diagnosis and treatment of non-muscle-invasive bladder cancer (NMIBC), however, radical cystectomy is the established treatment for muscle-invasive tumors. NMIBCs have a higher incidence of recurrence than muscle-invasive tumors due to conservative management. If left untreated or with improper management of NMIBC, its incidence of transformation into muscle-invasive tumors increases [4].

**Figure 14:**
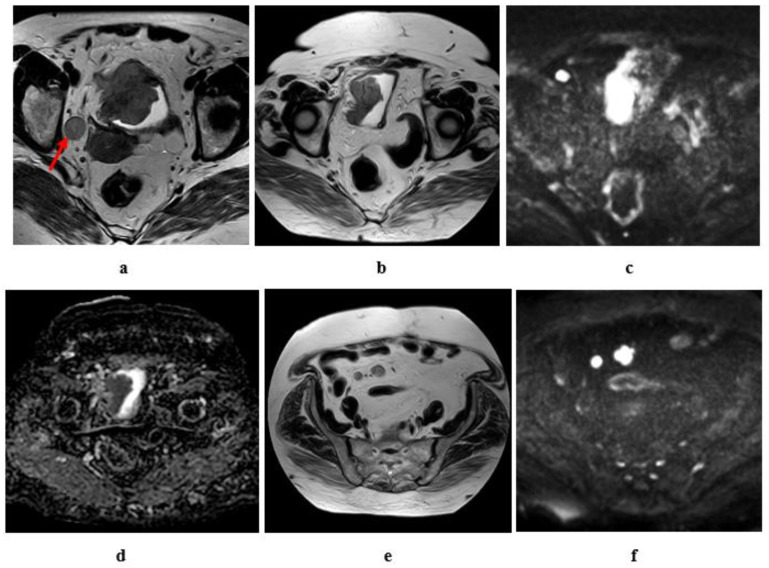
Tumor recurrence eight years after radical cystectomy and ileal-w-neobladder in a female patient, aged 67 years, the primary tumor was SCC, stage T3b N2 M0. (**a**) High resolution T2w MRI of the pelvis: an intravesical soft tissue mass at the right lateral bladder wall, RT side obturator LN (red arrow); (**b**–**f**) 8 years later (post radical cystectomy), (**b**) high resolution T2 w MRI of the pelvis: tumor recurrence in the ileal-w neo bladder, (**c**) diffusion w MRI (b value = 1400) revealed restricted signals of the recurrent tumor, (**d**) ADC of the mass with hypointense SI: 0.9 × 10^−3^ mm^2^/s, (**e**) high resolution T2 of the lower abdomen: two enlarged mesenteric LNs. (**f**) Diffusion w MRI ((**b**) = 1400): restricted diffusion of the mesenteric LNs.

Recurrence occurs after transurethral resection (TUR) of superficial bladder tumors in about 70% of patients [95]. However, in radical cystectomy with ileal-w-neo bladder, using a portion of the ilium, adenocarcinomas may develop due to intestinal mucosal irritation by prolonged contact with urine, which is considered as a secondary malignancy to primary urothelial cancer (Figure 14) [96].

## 8. Recent Advances and Future Directions

### 8.1. Multiparametric MRI

Several studies have assured that multiparametric MRI (mpMRI) is superior in the staging of bladder cancer, where morphologic (T2WI and DCE) and functional imaging such as DWI, diffusion tensor imaging (DTI) and perfusion-weighted are used in combination [48,97].

Differentiation of the normal bladder wall from the bladder tumor can be assessed on T2-weighted images [98], however DW images have a role in accurate assessment of muscle invasion as well as tumor grade [99].

### 8.2. Vesical Imaging-Reporting and Data System (VI-RADS)

In 2018, VI-RADS has been evolved to allow accurate implementation of MRI acquisition and interpretation for the staging of bladder cancer [100].

VI-RADS is a useful method for the prediction of muscle invasion using the anatomical category on a T2-weighted image (T2WI), the functional assessment with a diffusion-weighted image (DWI), dynamic contrast-enhanced (DCE) MRI and the overall VI-RADS score [101].

Applications of VI-RADS: VI-RADS score is best used prior to TURBT or intravesical Bacillus Calmette-Guerin (BCG). Therapeutic assessment can be achieved using combined preoperative MRI and VI-RADS scoring [100]. In patients with MIBC, MRI and VI-RADS scoring are of great importance in the tumor staging to assess the response from neoadjuvant therapy; to distinguish tumors to be managed with bladder-sparing surgery and chemo-radiation or TURBT [100]. In a recent study by Luo et al., they found that the VI-RADS score can be a useful image scoring modality for assessing the state of muscle invasion of bladder cancer with VI-RADS 3 or VI-RADS 4 as the cutoff value [102].

### 8.3. Positron Emission Tomography (PET)

The 18F-fluorodeoxyglucose (18F-FDG)-PET/CT is a functional method for assessment of the metabolic state of glucose intracellularly, via measurement of the standardized uptake value (SUV) maximum. Most cancers show increased uptake, with accurate detection of metastasis in small sized LN with increased metabolic activity, that are not suspicious by CT alone [103].

Many researchers reported that combined 18F-FDG PET/CT was superior in picking up distant metastasis in BCa [104].

FDG-PET/CT has a significant role in the accurate evaluation of tumor response to chemo-radiotherapy in patients with MIBC, and can select the appropriate time for operative management [105].

### 8.4. The Use of 18F- Sodium Fluoride PET/CT and Bone Scintigraphy

The positron emitting radiopharmaceutical agent 18F-Sodium Fluoride (NaF) is used for PET/CT for the detection of osseous metastasis and shows superior sensitivity with better image quality than bone scintigraphy in picking up the bony metastasis of bladder cancer [106,107]. Bone scintigraphy is recommended in cases with suspicious bone metastasis [7]. Precise preoperative bone scanning is correlated with increased survival of MIBC, and proper patient selection for surgical management [108].

### 8.5. Texture Analysis

Texture analysis is a component of computer aided diagnosis by quantitative measurement of the tumor heterogeneity and pixel distribution analysis. Entropy is a method for measuring the heterogeneity of the tumor and complexity of pixel intensity [109]. Many studies were done to assess its role in tumors of the genitourinary system, including renal and prostatic tumors for the accurate evaluation of the tumor grade and prediction of tumor recurrence [110].

High grade bladder cancers tend to have great values of entropy. It may be due to greater heterogeneity and aggressiveness of the tumor and direct tumor invasion of the surrounding structures associated with increased tumor angiogenesis and peritumoral edema [49].

Quantitative CT texture analysis has proved to have a significant role in discriminating the low grade from the high grade bladder cancer. Lower entropy signifies low-grade bladder cancer [111].

### 8.6. Radiomics

Radiomics is a technique where medical images are converted into high-dimensional data through the extraction of a large number of quantitative features, with sequential data analysis for decision-making using image SI, morphological, and textural features [112,113].

Up until now, radiomics have shown potential in bladder cancer detection, staging, grading, follow up and therapeutic response [114].

## 9. Conclusions

This article aims to serve as a concise review of the different current and advanced imaging modalities, used in the diagnosis and follow up of bladder tumors, however further studies are needed to assess their full potential and correlation with the pathological findings.

## Data Availability

Not applicable.
